# Intelligence

**Published:** 1827-12

**Authors:** 


					INTELLIGENCE.
monthly report of prevalent diseases.
JJURjNg fl
Weatlt ' S,eater part of the time comprised in our prespnt report, the
vvjjjj 61 las been remarkable for its mildness, but this has been accompanied
!nflti nillC'1 Wet aud with autumnal fogs, The prevalent diseases have been
by ibese circumstances, and affections of the digestive organs have
has Ver^.Comin?n' Diarrhoea, in particular, prevailed to a great extent, and
the illet'mes proved obstinate. Towards the latter part of the month, as
part.Ve<U'ler became colder, inflammatory affections became ir
? ' V Or tllP IV) nnniio ivinmKi<ntiA e\f flirt O 1 P_ r\Q6CO OTP C H I W 111
more numerous,
e*am U,lar'^ ?* tlie mucous membrane of the air-passages, of which some severe
P es have presented themselves to our notice.
Regulations for the Examination of Apothecaries.
T,{E Apothecaries' Hall; November 8, 1827.
bent"6 ^?nr* Examiners of the Society of Apothecaries feel it to be incum*
g?l "^?n *'lem to direct the attention of the medical profession to the u Re-
'"^pten'b ^?r "le ?xam'nat'on Apothecaries," published by them in
the p SL 'ie&u'at'ons, which are founded upon Reports annually presented to
Coiitin "t ^ss's,ants> whilst they pr?ve that the Court of Examiners have
Cation"6^ t0 ^'Ve '^eir serious consideration to the improvement of the edu-
expeli l'1C aP?thecary, point out at the same time those defects which their
<>ss /e"ce 'Ias shown to be of most frequent occurrence, and of the most
^ la importance. Of these, two may be more particularly alluded to:
J 8, Ni'w Series. 4 D
5(56 INTELLIGENCE.
the first is the want of a competent knowledge of the Latin language; the
second, the limited time too generally devoted to the various lectures and
other studies demanded by the Court; and both of these they have attempted
to remedy by requiring of all persons, who shall hereafter present themselves
for an examination, a more deliberate and methodical course of education.
The Court of Examiners desire that it may be recollected, that these Re*
gnlations are in conformity with the Act of Parliament " for better regulat-
ing the Practice of Apolhecaries throughout England and Wales; an Act*
which is in many respects imperative, in others discretionary, and in all of
such general application as to require if to be executed with a temperate and
steady attention to existing circumstances, combined with a due regard for
the welfare of the public^ and the progressive improvement of this branch of
the medical profession.
By order of the Court,
JOHN WATSON, Secretary?
Court of Examiners of the Society of Apothecaries.?Sir, As your Journal's
almost the only one in which the recent Regulations of the Court of Exam'*
ners of the Society of Apothecaries appear to be properly understood and
appreciated, and a s many of the medical periodicals have long continued to
indulge in the most unmeasured abuse of the Society of Apothecaries, as w?"
as of the Court of Examine rs, I have thought it proper to address a plaI"
statement of facts to you, in order that the public may perceive how ign?*
rant the majority of these Journalists are of the circumstances connected vr'1'1
the Act of Parliament " for better regulating the Practice of Apothecar?eS
throughout England and Wales," and that the members of the medical pro*
fession especially might know to what blind guides they have to trust, when
they depend upon such sources of information. It is true, indeed, that w',at
the authors of some of these publications want in knowledge, is amply st'p"
plied by malignity; a most palatable commodity in this age of intellect, a"
inore especially grateful when any public institution, or constituted autl'0*
rity, is to be calumniated.
The Society of Apothecaries, and the Court of Examiners, appear to '>e
accused chiefly of four great crimes:?1st, with compelling an apprenticesh'P
of five years; gdly, with refusing to acknowledge any foreign course
of study
as sufficient to entitle the'candidate to an examination; odly, with limit'0?
their admission of certificates to a few licensed teachers; and, 4thly, w'1'1
total incompetency to the task which they have undertaken to perform,''
namely, the examination of candidates.
In this enumeration, you will perceive that no distinction is made betwec''
the powers of the Court of Assistants, which is the executive of the Society 0
Apothecaries, and those of the Court of Examiners, whoare elected by then1'
though the latter had no hand whatever in framing the Act of Parlia?ne,,t'
and are bound to administer it as they find it. But the Court of Assistants ?
the Society are equally innocent of many, or most, of the provisions of
Act, and are, I make no doubt, perfectly ready to admit that some of lIS
clauses are imperfect, and that many ofessential importance might be added ?
the history of the Act will, however, show that this is not to be attributed 10
any fault ou their part.
In the year 1812; a public meeting of surgeon-apothecaries took place, f?r
Society of Apothecaries. 56?
^our5gSCS W'10"y foreign to the subject of medical education ; but, in the
attend ?' t''CSe "leetinSs> so interesting a topic could not fail to occupy their
"ien 10n>.anc' became a subject of lamentation that there was no power
Cation X'S^'!1? *? Prever|t any person, either with a very slender previous edi;-
an an ' i?r ,nf'ee^ without any education at all, from commencing practice as
_P?thecary.
jjj . ,Iesu't of these conversations was an application to the Colleges of
lherSIClanS an(^ SurS^ns, and to tlie Society of Apothecaries, to know whe-
I>(UI|.eit,l|er ot those corporate bodies would undertake to bring a Bill into
ti,?e fi'"11.11 to remedy this and other evils: each of them, however, at that
P'iecj eC Int^ t0 ^? S?' ant^ ass0c'at'011 ?f surgeon-apothecaries then ap-
think" ? "ie Soveinment upon their own account; but the Minister of the day,
te?t improper to establish a fourth Society, when three, fully compe-
ence "n^ertake the intended task of examination, were already in exist-
was '^''P'1^ to the Society of Apothecaries, who then undertook it. A Bill
aily cj ??^t into Parliament, which passed the House of Commons without
'he H USG w'Jatever relating to apprenticeship: this was afterwards added in
Peterh'186 ^o'ds, uPon l'ie motion of Dr. Parsons, the late Bishop of
,lgetl 0l0ugh, and finally carried, contrary to the wishes of the Society, who
jo . every e"deavour to prevent its adoption. It may, however, be justly
re |. C wbether the period of five years devoted to an apprenticeship is
be ? ?^jec^0,iable as it is now the fashion to represent it to be. It must
Cn^ol,ected that the prelate who first introduced this clause, and who
j u 11 as a most indispensable part of the Bill, was a man not only of
|,aij erary attainments, but also thoroughly acquainted with mankind; be
fully (. 1 '?n^ ''me keen master of one of the colleges at Oxford, and had
aild forV'DCed '1,mse'f ?f *'ie necessity of subjecting youth in all situations,
^eal ol' W'iatever employment they might be intended, to some temporary or-
effects Sa'"tary restraint, during which they might be taught to know the good
Vejlt ,S 11101'al and mental discipline. It may also be asked, what is to pre-
fesjj. >??th so situated from acquiring all the elementary parts of his pro-
Cvei|0na' education, including botany, the materia medica, chemistry, and
,lsiialIdnat0"1J'' d"rinS *'Je five>'eai's of his probation? a plan that is, in tact,
'''*rh| ^ ea'orced by all liberal practitioners, and the omission of which is
? y culpable. It is, however, only just to observe, that the Court of Ex-
,n e,s l|ave, upon more occasions than one, drawn the attention of both
tjCcsjCIS an(* apprentices to the necessity of employing the period of appren-
iir-'.'!' ln "le acquisition of professional knowledge.
(Jifgj11'1 resPect to the administration of tho Act, and the regulations for nie-
aiiri3- Cc'"cat'on, these are left to the discretion of the Court of Examiners,
it win I . * .
i, 11 1,6 seen, by a reletence to the Act, that some of the clauses being
a a lve? such as that regarding the age of the candidate, and the term of
te-U V'e^ not a^mit of any course of education excepting by particular
in ? e,rS' 01 *n Particular schools, is wholly false : on the contrary, they have
Z!tTiCeSh^ 'Iave 110 power to deviate from thein ; but, to assert
'eacl
111 each
have
*?? *vicigii auuuuio aim uuivci.-uicsj diiu navu uunc ou iui
su')stantial reason, tliat the Act leaves them (the Court of Examiners)
J,1('ge as to what are the proofs of a sufficient medical education.
ls equally untrue that the Court have limited the number of teachers of
ltav year granted certificates of qualification to several candidates who
studied in foreign schools and universities, and they have done so for
568 INTELLIGENCE. \
the various branches of study demanded by them : so far is this from'being
the case, that it will be found that, 111 consequence of thi% Act of Parliament,
and sanctioned by the approval of the Court of Examiners, anatomical and me-
dical schools have been opened in Manchester, Liverpool, Bristol, Birmingham*
and other large towns ; and it has not unfrequently happened that young men
have passed excellent examinations, who have commenced and completed
their medical education entirely in some provincial town. All that the Court
of Examiners have required is such testimony of the competency of the re-
spective teachers as they deem sufficient, and they have never been satisfied
without the most ample proofs.
Respecting the competency of the Court itself, it is only necessary to say
that their names are before the world, and they might triumphantly appeal
to the whole medical profession in support of their professional character,
either collectively or individually, were it necessary to do so ; and by tins
testimony they might be well contented to abide, flighting and despising, aS
it is quite evident they do, the aspersions of ignorance and malice, well aware
that, so long as they shall be compelled to reject nearly one-eiglitli of those
who present themselves for examination, they can be at no loss to account <or
having a multitude of enemies, and a consequent succession of misrepre-
sentations and slanders.
November 9th. OMICRON.
Observations on Dr. Gregory's Paper on Constitutional Inaptitude to C<"e'
Pox.?On reading the first article in your last Number, I could not but fee'
surprise at some of the questions put by Dr. Gregory respecting the doctii?e
of constitutional inaptitude to cow-pox, as he terms it; but I confess my aS"
tonisbment was not a little increased, when I had got through the concluding
paragraph. He there invokes the readers of your Journal to inform him (if
they have had the means of knowing,) " what were the peculiar opinions en-
tertained by Dr. Jenner"oii this subject? and adds, that he had in vain en-
deavoured to ascertain them. This strikes me as somewhat strange: I can
with difficulty persuade myself that a physician, placed in Dr. G.'s publ'c
situation, and desirous of that information which he now professes to seek
sedulously, could have neglected to search tor the opinions ot Dr. jenu^*?
touching this and other important topics in the history of cow-pox and ot
small-pox, contained in the first volume of the Life and Correspondence ot
Dr. Jenner, published several months since: therein he would have found the
instruction he requires, and much more. In the seventh chapter of that work
more especially, he would have seen such an affinity between the two affec-
tions elucidated, if not fully established, and that in entire conformity with the
opinion of Dr. Jenner, as might have satisfied his mind, as least so far as re-
garded the opinions of that illustrious man, who always considered small-P0*
and cow-pojc as modifications of the same distemper ; "and that, in emp'0}"1'
vaccine lymph, he only made use of means to impregnate, the constitution "it'1
the disease in its mildest, instead of propagating it in its virulent and conta-
gious form, as is done when small-pox is inoculated."*
Now, it was well known to Dr. Jenner and other medical men at this time>
that the constitution of many was at particular periods of life, and of so",c
* Seep. 241, Life of Edward Jenner, m,d. by Dr. Baron.
Inaptitude to Cow-Pox.?Pauper Lunatics. 569
throughout tlie whole of their lives, unsusceptible of smallpox infection ,
^erefore, a fortiori, "the inaptitude" might fairly be predicated of cow-pox >
,Independently of all individual or collective experience as to the reality.
Indeed, ** appears from several passages in the work just referred to, t lat
. :r* Jenner was so fully convinced of the affinity between, or rather of the
J entity of, small-pox and cow-pox as constituting one disease, that he thought
j! rpasonable to expect that the same general laws should govern both affec-
l0ns; not merely with regard to their prophylactic powers, but in many
other respects.
Having thus shown that Dr. Jenner's opinions have been before the public
?r several months in an authentic form, I must again express my surprise that
ley should have remained till this hour unknown to one who holds the highly
^sponsible office of physician to the Small-Pox and Vaccination Hospital in
^?ndon. It cannot ,lave arisen from a want either of interest or attention
!,lat he lias not even adverted to the work already mentioned ;? anil yet such
j* the case. In fact, the recorded doctrines of Jenner, (for they are more
an mere opinions,) the history of variola, and of the variolar vaccinae, loug it
orward so clearly in support of those doctrines, together with the strong and
^Ifhty arguments adduced by Dr. Jenner's biographer, particularly injis
sixtli? "j"" auuuucu "J - ? b--J > r ?
Gl e, ' anc' seventh chapters, seem either not to have met the eye of Dr.
. ?*' 0r> if they have, not to have made any impression on his memory.
^v|letjSto^e feared that the attention due to matters of such high import,
''ons M e '??k to l'ie 'iea't'1 an(* safety of the existing or of future genera-
It I)r' as n?t been sufficiently directed to this useful and instructive work.
a"cl n n 8 f? '',e statesman admirable lessons, drawn from historical research
tive i Ca' exPer'ence, for protecting the community from a most destruc-
before w'*''st to the medical man it opens clearer views than he ever
cni? enJ?yed of the nature and modus operandi of the great and almost mira-
U^us antidote.
eltci<ham ; Nor. nth, 1827. MEDICUS.
hte?V^er ^unat'cs-?T',e following notice, evidently published by authority,
1,^ aPPeared in the Times, being intended as an answer to the attack made
l'isd' '.'^erSeant Peu. on the College of Physicians, with regard to their ju-
Ctl?n ?ver mad-houses.
c. TO MR. SERGEANT PELL.
oir. Tn
?"ade'j Peec^ which is reported in the morning papers (o have been
^adv ^ ^?U yesterday, at a meeting of the Middlesex magistrates, you aui-
'aified ^ ?n W'lat y?n ale p'eased to term " an extraordinary opinion" enter-
povv ^ l'1C Colleee Physicians,?viz. that the commissioners have no
tjlat j^.to ref?sea licence; and I tliink it will be satisfactory to you to learn,
Wlios 1 t'Iere'n lhey are in error, they err with the late Sir Vicary Gibbs, to
large ?^lnion the members of his own profession, and the community at
Th V^6re heretofore accustomed to attach some little weight and authority.
(( 16 ?"?wing is a copy of his opinion:?
j^jce ^lc Opinion of Sir V. Gibbs, Attorney General, as to the Power of refusing
Petua^5 aPPears to n)e lhat the 14th Geo. III. c. 49, which is made per-
tly "a ^>th Geo. III. c. 91, leaves no discretion in the commissioners or
a'strates upon the granting of licences. They must grant them to all
570 INTELLIGENCE.
who apply, though they may have offended against the Act, and even been
convicted of such offences. The object of the legislature in requiring the
licences, was 10 bring all who kept houses of this description under public
notice, and to subject them at all times to inspection,
(Signed) "V. Gibbs."
" L. L., Nov. 29, 1810."
Whatever, sir, be the legal interpretation concerning the right to interfere
with parish paupers, 1 think you must be sufficiently aware, from a perusal
the evidence you quote, that the commissioners have never been wanting in a
watchful attention to the state of those unfortunate beings : that evideuce
contains repeated cases of interference with parish officers, on behall ot
pauper lunatics, and the minute-books of the commissioners abound with in*
stances of the like interposition.
When you say that " if the legislature had not given the College the povvc
to withdraw licences, they ought to have done to," to whom would you im-
pute blame ? Not to the College, surely, who at various periods have devoted
much time and trouble to a complete revision of the present Act, and had
every reason to expect that, long ago, the legislatuie would have sanctioned
an improved Bill, which, after much consideration, and many conferences wit'1
the late Mr. George Rose, was carefully drawn up by Mr. W. Harrison. Tl?s
Bill was rejected by one branch of the legislature; but assurances were afte?'
wards given by the highest legal authority, that an enactment, in which evevy
objection should be provided against, would be framed without delay. 'l'.
then, fair or candid to reproach the College of Physicians with a delay,
which assuredly they are not ill any degree the cause?
Did not the committee of the House of Commons report in 1815, " t'13'
some new provision of law is indispensably necessary?'' and are those whose
only business it is to carry such improved law into effect to be censured be-
cause it is not yet in existence? With means confessedly inadequate, I C?1V
tend that much has been done to better the condition of lunatics.
Recollect only what was the state of those receptacles of misery before tl??
commissioners' visitations were enforced : the most culpable facilities 0
admission,?the grossest neglect after admission,?foul air, scanty food, 10
say nothing of the substitution of chains for personal attendants. AltH?u?
the Act, for reasons best known to those who framed it, has excepted tU<j_
names of paupers from being returned to the commissioners, the eye
watchfulness and pity never failed to be turned towards these wretched 'jC
nigs; and the result has been greater care, larger space, belter ventilation a
more complete separation of the sexes, and a much milder mode of restrain1*^
I do not venture to impugn the motives, or to question the benevolence*
those who have so strenuously advocated the erection of a county asylum; l)llt
I do think myself fully warranted in repelling that obloquy so unjustly thro*ul
on the commissioners, and in asserting, with confidence, that asmuchhas beetl
done by the College of Physicians to improve the condition of these houses a'
it was possible for any body of men to do with the very limited powers C?D
fided to them.
Iam, sir, your humble servant,
Nov. 16. M.
The first part of this statement is perfectly satisfactory, and dearly prove5
the very limited powers of the commissioners; the second part see?,s
List of Books. 57V
is to? Cia'n, tlie merit of li:iv ing done that which the purport of the document
Prove they had not the power to do!
low-g.'rf ^ew Allows and Licentiates of the College of Physicians.?Fel-
AltJe,. r" ^rancis Bisset Hawkins, Golden-square ; Dr. William Babington,
^anchlan^ "^' ^r' ^?c'iart' Fonnby, Liverpool; Dr. George Freckleton,
Lj Ser> a"d Dr. Edward Seymour, George-street, Hanover-square.
PianCeii,'ateS! ^r' Francis Henry Ramsbotham, New Broad-street; Dr.
fhonC1S ^ann? Montague-street, Portman-square; Dr. Charles Willard
street^r* ^ ^'"'ain Byam Wilmot; Dr. John Barclay Sheil, St. James's-
sfreet-' P' ^enry Kiley, Great Ormond-street; Dr. Marshall Hall, Keppel-
Dav; i'i, r* Carles Maxiraillian Kind, West-street, Finsbury Circus; and Dr.
1(1 Barry.
In #
tj 3 seventeen physicians became Licentiates : in 1827, only nine.?
le College look to the signs of the times.
METEOROLOGICAL JOURNAL,
From October 20th, to November '20th, 1827.
liy Messrs. Harris and Co. Mathematical Instrument Makers, 50, High Hoi born-
20
21
22
23
24
25
20
27
28
29
30
31
Nov.
1
2
3
4
5
6
8
9
10
11
12
13
14
15
1(1
17
18
19
10)
.53
o
Thermom.
55 61
Barometer.
29.57
29.37
29.07
29.29
29.84
29.97
29.89
29.37
29.49
29.90
29.01
29.70
29.85
29.99
30.00
30.15
30.27
30.13
30.05
20.9-1
29.07
29.90
29.87
30.05
30.03
29.83
29.47
29.55
29.77
30.0 >
80.11
De Luc's
Hygrom
99
98
100
90
95
99
8(5
99
99
80
97
70
89
87
91
98
97
90
97
96
98
100
91
90
97
80
100
100
100 loo
100 100
100 100
Winds.
WSW
N
SSE
SVV
SE
SW
ssw
SE
E
NNE
V/NW
NNE
N
NNE
WNVV
I vv
i \v
WNVV
WNVV
SE
NW
NW
1 NW
i N
| NW
SB
I S
1 SE
i E
! SE
SSE
N
SE
S
SE
WNW
SW
S
WNW
ENE
NNE
ENE
N
WNW
N
W
W
w
NW
ESE
NE
NW
WNW
NW
WNW
NNW
S
ENE
SSW
s
SE
SE
Atmospheric Variation
9 a.m. 2p.m. 10 P-'n
Rain
Fosgy
Rain
Foggy
Ham
Fair
Cloudy
Rain
Fair
Cloudy
Fair
Cloudy
Foggy
Overca.;
Cloudy
Fair
Foggy
Sleet
Kaiu
Foggy
Fair
llain
Fair
Rain
Fair
Fougy
Rain
Fair
Cloudy
Rain
Fair
Foagy
Fair
Fair
Rain
Cloudy
Sleet
Clou'ly
Fair
Cloudy
Sleet
Foegy
Cloudy
Sleet
Fine
Sleet
Cloudy
Kaiu
Fair
Sleet
Fair
The quantity of Rain fallen in the month of October, was 3 inches and 31.100ths

				

